# Homogeneity of Electro-Mechanical and Optical Characteristics in Ring-Shaped MEMS Shutter Arrays with Subfield Addressing for Interference Microscopy

**DOI:** 10.3390/mi16020168

**Published:** 2025-01-30

**Authors:** Philipp Kästner, Basma Elsaka, Mustaqim Siddi Que Iskhandar, Steffen Liebermann, Roland Donatiello, Shujie Liu, Hartmut Hillmer

**Affiliations:** 1 Institute of Nanostructure Technologies and Analytics (INA), Technological Electronics Department and Center for Interdisciplinary Nanostructure Science and Technology (CINSaT), University of Kassel, Heinrich- Plett-Straße 40, 34132 Kassel, Germany; elsaka@ina.uni-kassel.de (B.E.); liebermann@ina.uni-kassel.de (S.L.); donatiello@ina.uni-kassel.de (R.D.); shujie.liu@ina.uni-kassel.de (S.L.); hillmer@ina.uni-kassel.de (H.H.); 2 Nanoscale Glasstec GmbH, Heinrich-Plett-Str. 40, 34132 Kassel, Germany; mustaqim.iskhandar@nanoscale-glasstec.com

**Keywords:** optical MEMS, micro-iris, interference microscopy, actuation voltage, response dynamics, transmission, circular shape, curvature, radial and angular homogeneity

## Abstract

We present a MEMS array-based approach for micro-irises called “ring shutter”, utilizing subfield addressing for applications in advanced micro-optics, such as interference microscopy. This experimental study is focused on investigating the homogeneity of electro-mechanical and optical characteristics within and between subfields of a lab demonstrator device. The characterization aims to ensure crosstalk-free and swift optical performance, as demonstrated in a previous study. For this purpose, the transmission in the initial state, actuation voltages, and response dynamics are measured for each electrode and the entire device, and the results are thoroughly compared. The measurements are conducted by expanding an existing optical actuation setup via tailored 3D-printed apertures, to isolate selected rings and zones. Evaluation of measurement data confirms the stable and crosstalk-free operation of the ring shutter. Both angular and radial homogeneity are robust and follow the expectations in the experiment. While transmission, actuation voltage and closing time slightly rise (up to 25%) with increased radial position represented by five discrete ring sections, the characteristics for different angular zones remain nearly constant. Response times are measured below 40 µs, actuation voltages do not exceed 60 V, and the overall transmission of the ring shutter yields 53.6%.

## 1. Introduction

**Figure 1:**
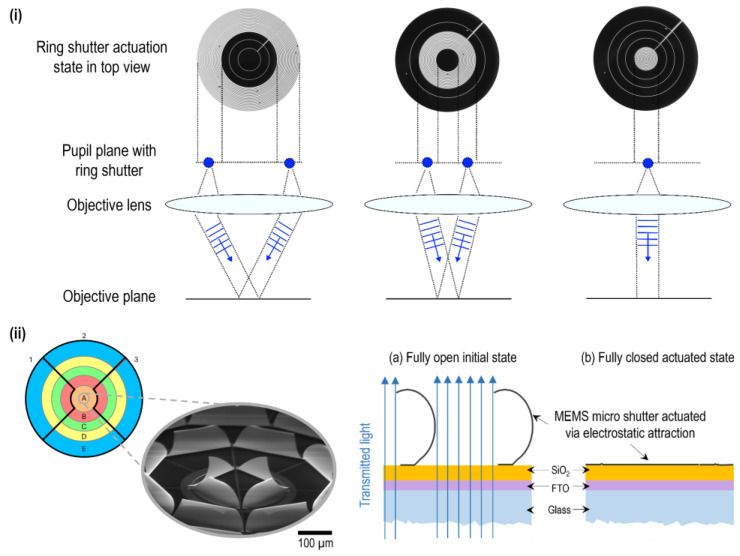
(**i**) Ring shutter in different actuation states applied as a light manipulator for control of incident angle, e.g., for coherence scanning interferometry. The depicted states are switched within several µs using a multichannel waveform generator. (**ii**) Schematic illustration of the ring shutter with an SEM micrograph of the thoroughly fabricated inner ring A in the initial state as an inset. To the right, a cross-sectional view of individual MEMS shutters illustrating the layer system and bistable actuation states “open” and “closed”.

Micro-irises (or, more technologically, miniaturized aperture diaphragms) are desirable for a variety of optical systems and implemented via multiple different technologies and approaches, including optofluidics [1,2,3], electrochromism [4], liquid crystal elastomers [5,6] or MEMS [7,8]. A recent publication [9] discusses using circular and ring apertures to manipulate the incident angle of illumination light in the context of coherence scanning interferometry, similar to Figure 1i. This methodology can be used for challenging measurements, such as samples with mesa structures. It modifies the interference signal in shape and spectrum, ultimately enhancing the signal quality and the signal-to-noise ratio. To enable dynamic measurements, the mechanical apertures can be replaced by a micro-iris with advanced spatial controllability, such as individual annuli sector or ring actuation (see Figure 1i). For this purpose, a MEMS array-based approach for an advanced micro-iris, called “ring shutter”, is developed and studied in a collaboration project at the Institute of Nanostructure Technologies and Analytics (INA) (see Figure 1i). The ring shutter is based on the platform technology of micro-mirror arrays [10]. Recently, the first lab demonstrator and its convincing overall characteristics were presented [8,11]. Compared to the other approaches, the electro-mechanical performance is strong, while the optical performance offers potential for improvements. The unique characteristic of the device is the capability for subfield addressing, allowing spatially controllable actuation states and flexibility in design and dimension. Figure 1ii introduces the layout of the demonstrator and elaborates the working principle. The MEMS elements adhere on a glass substrate covered with a transparent conductive oxide (TCO; here, fluorine-doped tin oxide (FTO)) and an insulating layer (here, SiO_2_). After fabrication and in initial state, these so-called micro shutters are curled out-of-plane—shown in SEM micrograph in Figure 1ii—allowing light to transmit while applying a voltage to the TCO with respect to the metallic shutter above the actuation threshold leads to the pull-in of the element. Hence, it closes, and the light cannot transmit anymore (is blocked) as long as the voltage is applied. The ring shutter demonstrator consists of five individual rings A–E filled with MEMS indicated with colour in Figure 1ii and is divided into three zones via a structured TCO layer, represented in the same schematic drawing with black lines. The total device diameter is 18 mm.

**Figure 2:**
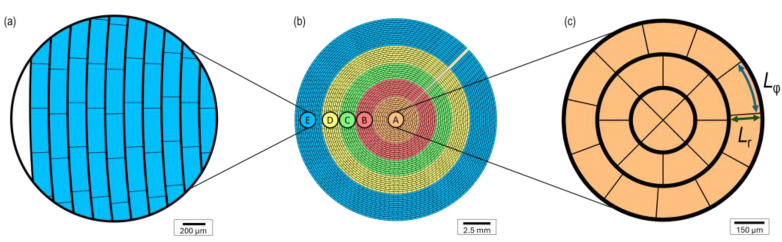
(**b**) Schematic overview of the MEMS elements design of the ring shutter in top view. (**a**) and (**c**) Insets of the outer edge (radial coordinate *r =* 9 mm) and centre (*r =* 0 mm) displaying the last ten (**a**) and the first three (**c**) subrings. The MEMS shutters are electronically connected but mechanically decoupled via an opaque metal grid visible as black gridlines in the subfigures. Therefore, one cell of the grid resembles one element. All illustrations are drawn to scale. The aimed size of an element is *L*_r_ × *L*_φ_ = 150 µm × 400 µm, theoretically reached at infinite radius *r*.

As a consequence of the circular shape of the iris, the micro shutters’ individual geometry is similar but not identical. Figure 2 shows a schematic overview of the top electrode built by the MEMS elements in top view as well as the two insets of the extreme cases of radial position *r*: The centre (*r =* 0 mm) holds triangular and strongly curved trapezoids, while the elements at the outer edge (*r =* 9 mm) are nearly rectangular. In contrast, there are no changes in geometry along the angular direction. Note that the zones are not depicted, as they are on the bottom electrode formed by the structured TCO. Hence, they are also not visible in the final fabricated device shown in Figure 1i. The aimed size of the MEMS elements is *L*_r_ × *L*_φ_ = 150 µm × 400 µm. The radial length *L*_r_ is fixed. The angular width *L*_φ_ satisfies the boundary condition that the circumference at radius *r* must be an integer multiple of the constant angular width *L*_φ_ within one subring, leading to minor deviations in the range of 2% between subrings, excluding the first six.

From previous experimental studies and simulations [10,11,12], we know that geometrical variations in the MEMS shutters influence their characteristics and can be used to tailor the overall device to specific applications. Hence, we expect a high homogeneity of properties along the angular direction, whereas the radial component might offer interesting trends. Furthermore, the micro shutters are organized and actuated in subfields (rings and zones), which can induce inhomogeneities to some degree and need to be investigated [13]. Therefore, this experimental study focuses on the homogeneity of ring shutter properties, namely the transmission in the initial fully open state, the actuation voltage and the response dynamics (i.e., closing and reopening times) within and between the respective rings A–E and zones 1–3 in comparison.

## 2. Materials and Methods

### 2.1. Fabrication Process

The fabrication of the ring shutter employs standard MEMS processes. It begins with a 2.3 mm thick glass substrate coated with a 650 nm layer of fluorine-doped tin oxide (FTO), which serves as a conductive bottom electrode. To enable the feature of subfield addressing in zones, the FTO layer is sub-structured through an etching process. Subsequently, a 1 µm thick SiO_2_ isolation layer is deposited on the substrate using plasma-enhanced chemical vapour deposition (PECVD). The next step is optical lithography creating the design of the ring shutter array, which also functions as the top electrode. The lithography process includes the use of a special bi-layered photomask which facilitates contact and proximity lithography in a single step. Afterwards, a multilayer metal system, consisting of (Al-Cr-Al) with thicknesses (90–15–67) nm, respectively, is deposited via electron beam physical vapour deposition (EBPVD) to achieve the ideal radius of curvature (RoC) in curling of the MEMS elements after being released [14]. The photoresist is then removed through a wet etching process using a remover bath at an elevated temperature of 80 °C (hotplate) for 18 h, allowing the ring shutter elements to stand freely out of the plane into the initial open state. Finally, the sample is dried by transferring and heating it inside an isopropanol bath on a hotplate at 55 °C and subsequent evaporation of solvent at clean room atmosphere (laminar airflow) after takeout of the bath. As a last step, the ring shutter is housed in a nitrogen atmosphere, ensuring the humidity level is maintained below 4%. Indeed, residual humidity in the atmosphere might lead to the adsorption of water molecules on the substrate surface, inducing stiction of the MEMS elements to the insulating layer (substrate) through capillary force, especially when the substrate is cool. For this purpose, residual humidity may be kept as low as possible, and housing is performed at elevated substrate temperature.

### 2.2. Methodology of Characterization

**Figure 3:**
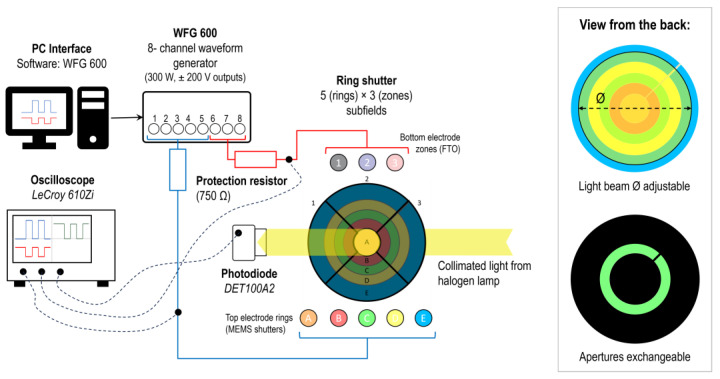
Schematic overview of the optical actuation setup. The ring shutter is actuated using an eight-channel waveform generator and is illuminated by a collimated light beam from a halogen lamp, which passes an exchangeable 3D-printed aperture depending on the investigated ring or zone (see Figure 4). The light is collected with a photodiode. Both the resultant collected light signal and the applied waveform are monitored and tracked on an oscilloscope.

Figure 3 shows the characterization setup of the ring shutter with respect to actuation voltage, response dynamics and transmission in the initial state for each ring (radial steps) and zone (angular steps), individually. For this purpose, a photodiode (Thorlabs GmbH, Dachau, Germany, DET100A2) measures the light intensity incident from a collimated beam generated by a colour-neutral halogen lamp, transmitted through the ring shutter and monitored with an oscilloscope (Teledyne LeCroy, Heidelberg, Germany, WaveRunner 610Zi). To enhance the signal-to-noise ratio of the individual subfields, the selected ring or zone is isolated through a corresponding aperture aligned on the back of the device, see Figure 3 on the bottom right for ring C, exemplarily. Furthermore, the light beam diameter *Ø* is adjustable to enhance the light intensity for inner rings. The device is then actuated using a software-programable eight-channel waveform generator (FLC Electronics AB, Gothenburg, Sweden, WFG 600-8) to close and reopen selected subfields independently with an adjustable protection resistor of 750 Ω connected in series for each of the eight channels, illustrated in Figure 3. For example, to obtain the closing and reopening of a selected ring (e.g., C) solely, a potential difference well above the closing threshold (i.e., the fully closed state of the MEMS elements, which in this case defined at 80 V) is applied to the selected top channel (+40 V) with respect to all three bottom channels (−40 V) while the non-selected rings are put to ground. This bipolar actuation minimizes crosstalk issues in case of complex subfield addressing actuation scenarios since non-selected subfields are partially actuated (±40 V), but well below the threshold and therefore not pulled in, while the selected subfields are subjected to the full actuation voltage.

Static actuation, i.e., holding a steady closed position of MEMS elements in the targeted subfield, is achieved via DC pulses, whereas dynamic actuation requires customized, advanced waveform designing which is addressed in the corresponding sections. The applied voltage is monitored using the same oscilloscope (LeCroy 610Zi), enabling the tracking of applied voltage and transmission simultaneously. With this, statements about the in situ actuation state of the MEMS elements can be made, e.g., closing threshold (closing begins), fully closed state, reopening threshold and fully opened state as well as ringing. In the beginning of the experiments, no voltage is applied to the device to measure the transmission in the fully open initial state. After that, a low-frequency bipolar sinusoidal wave is used to identify the actuation voltage via the transmittance-voltage curve (T-V). Lastly, applying a step function allows the analysis of the response dynamics.

**Figure 4:**
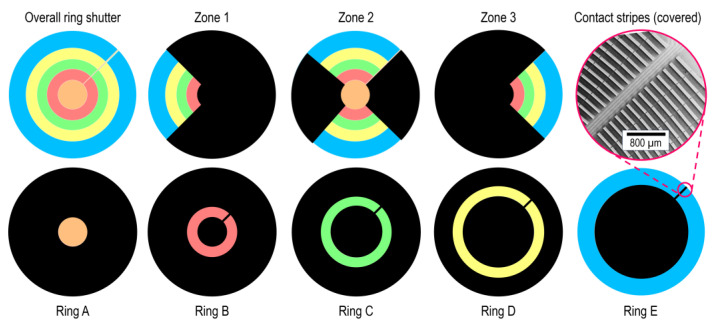
Illustration of the 3D-printed apertures used for the developed measurement methodology, resembling the zones 1–3 as well as the rings A–E. The measurement for the whole ring shutter (overall) where no aperture is applied is used as comparison. Note that in each case, except the overall, the contact stripes visible on the inset at the top right are covered.

The 3D-printed apertures visible in Figure 4 have been fabricated using a filament printer (Prusa Research a.s., Praha, Czech Republic, MK4) with non-transparent PLA material. The thickness is 2.5 mm, and they have been tested for sufficient optical density. The apertures have been assembled in the optical path on the back of the sample using a holder (at 1 mm distance to the bottom of the substrate). The accuracy of printing dimensions is specified at ≤100 µm. To ensure the fit onto the desired ring/zone, the cutouts are designed with an offset of 200 µm smaller than the actual dimensions of the ring/zone.

## 3. Results and Discussion

We are evaluating the following measurement data regarding homogeneity within the ring shutter:

- Transmission in the initial fully open state for control of optimized stress-induced curling.
- Transmittance-voltage (T-V) curve for determination of actuation voltage and prevention of crosstalk.
- Response dynamics (closing and reopening speed) for targeted high frequency applications.

The fabricated ring shutter has a pixel error rate of 0.7%, stating that 99.3% of MEMS elements are fully functional and therefore suitable for measurement and evaluation.

### 3.1. Transmission in the Initial Fully Open State

Initially, no voltage is applied to the device, which exhibits its fully open state. By exchanging and aligning the ring/zone apertures on the back of the ring shutter using a holder (as well as adjusting the collimated beam diameter of the source), the light intensity with and without (as reference) ring shutter is measured for each configuration presented in Figure 4. The corresponding transmission is then calculated as ratio between both values, each. Additionally, the relative deviation in the transmission value of each ring/zone with respect to the overall device is determined. Table 1 shows the summarized results.

**Figure 5:**
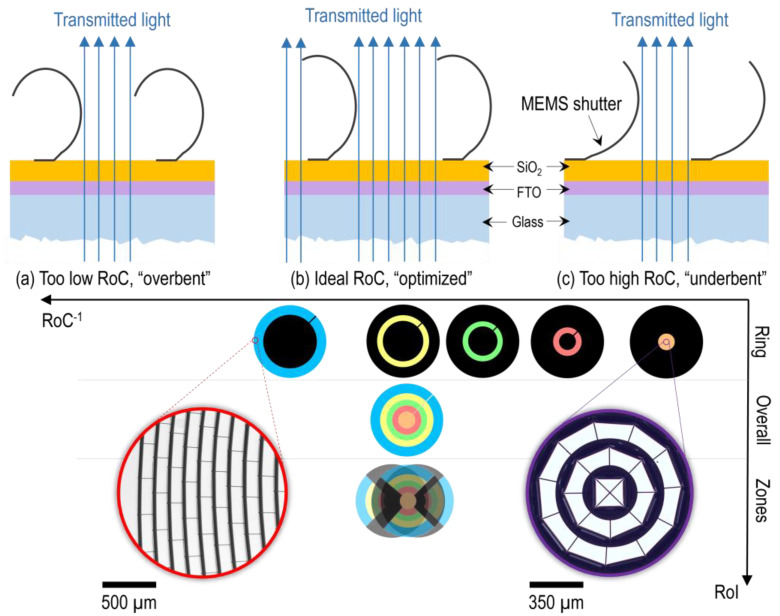
Mapping the region of interest (RoI), i.e., rings A–E, to the radius of curvature (RoC) observed in micrographic investigation supports the explanation of the transmission findings. The schematics on the top part of the figure depict different situations of illumination due to the given RoC of the freestanding micro shutters: (**a**) Overbent micro shutters with lower RoC, (**b**) optimized/ideal RoC allowing maximum transmission, and (**c**) underbent micro shutters with higher RoC. The graph at the bottom part of the figure shows the distribution of the resulting transmission measurements, which are in accordance with the qualitative rating of the RoC. The two micrographic insets are taken in top view with back illumination.

The overall transmission of the ring shutter is measured as 53.6%. The individual rings deviate in the range of –17% for the centre ring A to around +8% for the outer ring E with respect to the full device, increasing ring by ring. In contrast to that, the zones show only a slight deviation to the reference. The latter result supports a stable fabrication with homogeneous stress distribution in different directions, while the first phenomenon is explained with the observed radius of curvature (RoC) for the out-of-plane curled 3D microstructures discussed in Figure 5. Since the distance of the photodiode to the ring shutter is roughly 40 cm while the total diameter of the device is 18 mm and the height of the MEMS elements is maximum 150 µm, the measured transmission corresponds to the projection of the 3D microstructures on the glass substrate visible when taking micrographs with back illumination in top view (see Figure 5). The contribution of scattered light is negligible. Therefore, the transmission connects to the RoC of the MEMS elements, defining the overlap with the opaque hinge and the transparent gap between them (which is the channel for transmitting the light, see the cross-sectional subfigures in Figure 5).

As clearly visible in the two micrographs from the centre and outer part in Figure 5 bottom, the curling is optimized for ring E, minimizing the overlap with the transparent part while the elements in ring A are underbent. The reason for the difference between the two extremes lies within the geometry of the hinge side. With increasing radius, the (triangular) elements form first a strongly curved trapezoid, and finally approximate a rectangular shape. As shown in the micrographs, a greater curvature of the hinge side towards the centre more effectively suppresses mechanical curling, and therefore, the measured transmission value is lower. Since the weight of the individual rings to the overall measure is different (decreasing from E to A), the dominant ring E is chosen to be at the edge of overbending, yielding an overall device optimized in terms of transmission.

To further enhance the transmittance of the ring shutter, various methods might be considered. Proposed actions refer to modifications in the substrate system (glass substrate with FTO and insulating layer), the active layer, i.e., the MEMS elements, and the top cover glass. Considering that the current substrate system has a measured transmission of 85.8% and the top cover glass yields 90.0%, the transmission of the active layer returns 69.4% in a simplified calculation without consideration of multiple reflections. This means that there is still potential for improvements in each part, especially the active MEMS layer itself. Methods of improvement include (i) the change in transparent bottom electrode material from FTO to ITO as well as the deposition of an anti-reflection layer on the bottom of the glass substrate, (ii) the reduction in metal grid density and planarization of currently curled MEMS elements, and (iii) the reduction in top cover glass thickness, together with anti-reflection coatings on both sides. Based on the measurements of alternative substrate systems and top cover glasses, along with advancements in micro-mirror platform technology [10], for the ring shutter, a maximum transmission value exceeding 70% is expected in the near term.

The transmission in the fully closed state is measured as around 2% for the overall ring shutter. The transparent isolation gaps in between the rings and contact stripes which make up 1.6% of the total device area, contribute to this value. For the individual rings/zones it is significantly lower (<<1% in case of a defect-free subfield), but strongly dependent on the number of defects in the studied subfield. Given the random location of defects, the transmission value in the closed state does not directly reflect the homogeneity between rings or zones when residual defects are present.

### 3.2. Transmittance-Voltage (T-V) Curve

**Figure 6:**
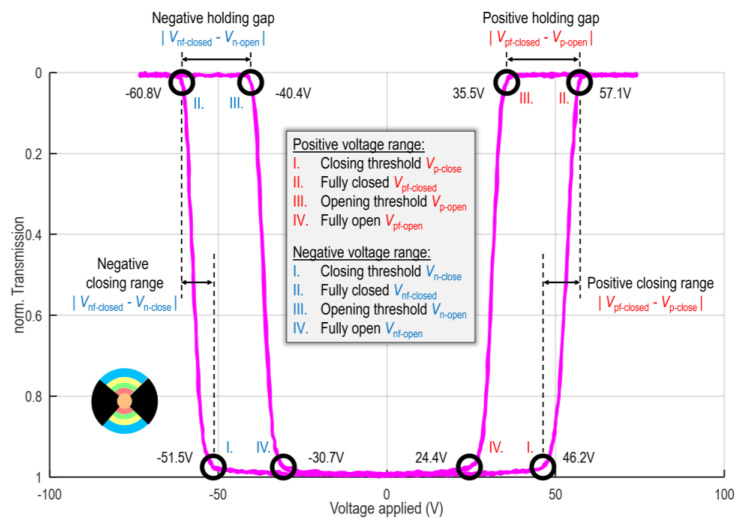
Transmittance-voltage (T-V) curve for zone 2. The applied voltage is on the horizontal axis, and the normalized transmission is on the vertical axis. The resulting hysteresis curve traversing the characteristic points of the I. closing threshold, II. fully closed state, III. opening threshold and IV. fully open state of the micro shutters is observed in both the negative and positive half cycle of the bipolar sinusoidal wave. With this, the closing range and holding gap can be defined.

A low-frequency bipolar sinusoidal wave with an amplitude of 76 V is applied between the top and bottom channels of the selected ring/zone, while the photodiode measures the corresponding transmission by the correct choice of ring aperture and light beam diameter, simultaneously. Since the MEMS elements follow the pulse quasi-instantly (µs reaction time vs. ms pulse steps), the actuation state can be directly connected with the present voltage by analyzing the T-V curve. Figure 6 shows a typical T-V curve of zone 2 as an example. The transmission is normalized, i.e., a value of “0” refers to the fully closed state, whereas “1” represents the fully open initial state. For the positive half-cycle (notation index *p*) of the sinusoidal wave: The curves begin at the lower centre until the elements are beginning to close at a certain closing threshold *V*_p-close_ and finally reach the fully closed state (transmission value is 0) at *V*_pf-closed_. When the applied voltage is decreasing again, the MEMS elements stay fully closed even at reduced voltage due to the minimum distance between the electrodes (holding gap), until they are starting to be pulled-out at the opening threshold *V*_p-open_ reaching the fully open state again at *V*_pf-open_. This procedure is then repeated for the negative half (notation index *n*) of the bipolar sinusoidal wavefunction resulting in a double-sided hysteresis curve (see Figure 6). Using this curve, the closing and reopening thresholds, as well as the fully closed and fully open states, can be identified as 2.5% and 97.5% signals, respectively.

The positive/negative closing range is defined as the absolute difference between voltages for the fully closed state and the closing threshold for both polarities in a subfield. It describes how homogeneous the MEMS elements within one ring/zone (e.g., zone 2 in Figure 6) close. The smaller this value, the less differences between the shutters in terms of pull-in behaviour.

The deviation in positive/negative closing threshold is defined as the ratio of closing threshold between the selected ring/zone and the overall device. It determines the homogeneity of actuation voltage between different rings/zones. In this regard, a small value is attractive since crosstalk is reduced and prevented.

As the last critical parameter, we define the holding gap as the absolute difference in applied voltage between the fully closed state and the (re)opening threshold within the selected ring/zone. It describes how much the applied voltage can be effectively reduced without letting the selected shutters reopen (after they reached the fully closed state), i.e., to still hold them in closed state. The larger the value, the higher the possible voltage reduction after pull-in to reduce the load on the insulating layer and enhance its lifetime. Furthermore, potential crosstalk, which might arise during actuation due to finite (not small) deviation in the closing threshold between the subfields and the large closing range of the selected subfield, can be cured. In other words, the subfield addressing in the ring shutter application aims for a low closing range, low deviation in closing threshold, moderate/high holding gap, and high symmetry between positive and negative half-cycle (±values in the table are as similar as possible) for each ring/zone individually as well as in comparison.

Table 2 shows the evaluation results. The positive and negative measurements are directly summarized and indicated in red and blue, respectively. The terms in the table are introduced previously in Figure 6 and the main text. A positive deviation in the closing threshold means that a higher voltage with respect to the whole device is required.

As can be seen in Table 2, the symmetry between the positive and negative half cycle is high, with a tendency to lower required voltages in the first positive half. The closing range is very consistent, ranging from around 8 V to 11.2 V maximum for ring D in the negative half, while the holding gap exceeds by a factor of two for nearly every ring and zone. The deviations in the closing threshold are typically within 3%, except the positive half-cycle in rings B–D. Interestingly, the rings slightly increase in closing threshold from inside to outside (A–E), suggesting that the curvature influences the effective closing threshold of the elements. The stronger the curvature, the less voltage is required. This is consistent with the micrographs in Figure 5, indicating that the elements in ring A are less curled and, therefore, closer to the counter electrode. Again, the zones are very consistent with the overall measure. In summary, the T-V measurements are highly homogeneous, and support the crosstalk-free operation demonstrated in [8].

### 3.3. Response Dynamics (Closing and Reopening Speed)

**Figure 7:**
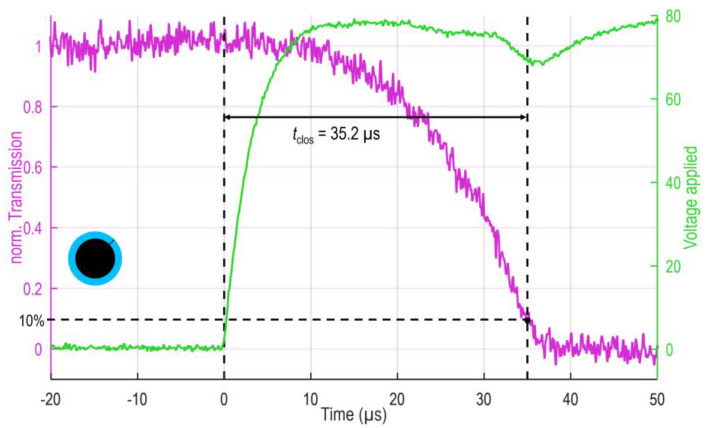
Closing dynamics of ring E. The photodiode signal is drawn in magenta, while the applied voltage is indicated in green. The finite rise time of the applied voltage results from the protection resistance, the drop indicates the fully closed state of the elements. For the evaluation of closing time *t*_clos._, the 10% threshold is considered.

Finally, a rectangular step function with a step width of 500 ms and amplitude above the actuation voltage (here, 80 V) is applied to the device, and the transmission of the ring/zone is measured simultaneously within ±100 µs before and after the step occurred. The resulting transmittance-time (T-t) curve captures the closing and reopening dynamics of the ring shutter. The threshold is set to 10% and 90%, respectively. Figure 7 shows the closing at the example of the outer ring E of the device, with the diode signal displayed in magenta and the applied voltage in green. Due to the protection resistance of 750 Ω, the voltage curve depicts a finite rise time (around 10 µs) influencing the measurement. Since this is the case for all the following measurements, the relative comparison of overall, ring and zone is still valid. Interestingly, the applied voltage drops again once the diode signal approaches zero, indicating that the MEMS elements are completely pulled in. Indeed, the distance between electrodes has continuously decreased from around 5 μs (closing threshold ~50 V is reached) with increasing slope (speed). When the elements are close to hitting the surface, this results in the maximum reduction in distance to the other electrode per time (equivalent to the most substantial capacitance change rate). Hence, the temporal position of the voltage dip gives rise when the fully closed state of the elements is reached. The evaluated response times are listed in Table 3.

The closing completes within 35 µs for all rings and zones. The response time is rising with increased radial position, i.e., ring A closes the first and ring E the last, which is consistent with the findings for closing threshold and transmission (RoC). The overall deviation is within ±20%. It is worth mentioning that the full device measurement resembles the average of all rings weighted with their effective area. Once more, the zones do not show significant variations.

**Figure 8:**
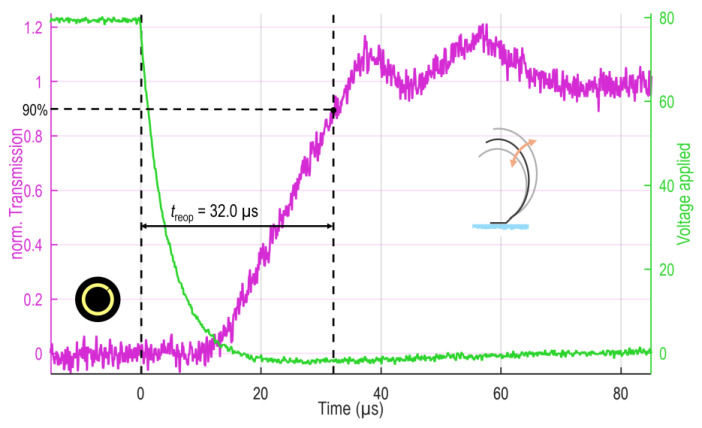
Reopening dynamics of ring D. The photodiode signal is drawn in magenta, while the applied voltage is indicated with green. The finite fall time of the applied voltage results from the protection resistance. The MEMS elements show a strongly damped oscillation (ringing). For the evaluation of reopening time *t*_reop_., the 90% threshold is considered.

While the closing is achieved by electrostatic force rapidly exceeding mechanical counterforces and, therefore, abruptly pulling the MEMS elements in, the reopening solely relies on elastic restoring and inertia, leading to differences in movement and response time. Indeed, the reopening dynamics show a strongly damped harmonic oscillation (ringing), as shown in Figure 8 above *t* > 25 µs for ring D as an example. This time, a finite fall time of the applied voltage of around 10 µs is introduced to all measurements by the protection resistor valued at 750 Ω, leading to a delay in the MEMS shutter response (see Figure 8).

As can be identified in Table 3, the trends in the reopening response are not as clear as in the case of closing due to the purely mechanical driving force and the occurring oscillation. Individual rings tend to reopen faster, and zones behave similarly. Absolute deviations lie within 8 µs. The overall device as the sum of the rings (or the zones) reaches the 90% threshold within 40 µs.

To conclude, the response dynamics are fairly homogeneous and reasonably fast, at less than 40 µs. Both closing and reopening responses are tailored by design modifications [10,12]. Additionally, the response times can be further decreased by either increasing the voltage amplitude of the waveform or decreasing the protection resistance. Based on the swift response of the ring shutter, actuation frequencies above 5 kHz can be applied.

## 4. Conclusions

In this study, a MEMS array-based approach for advanced micro-irises called ring shutter was introduced, and a lab demonstrator thoroughly characterized with respect to its homogeneity in transmission in the initial fully open state, actuation voltages and response dynamics. For this purpose, an existing optical actuation setup was expanded by tailored 3D-printed apertures, allowing the isolation of selected rings and zones of the device. The measured transmittance-voltage (T-V) curve gave rise to a high degree of homogeneity in the actuation voltages, supporting the crosstalk-free operation demonstrated in a previous study. Despite the consistent behaviour of the different zones observed, the rings showed a clear trend of slightly increasing closing threshold (up to 7%) with increasing radial position. The same was identified in the transmission measurements (up to 25%), which could be related to the curling RoC of the MEMS elements and the resulting projection on the substrate plane in the top view. While the closing times also followed this trend (deviations within ±20% to the overall device), the reopening times were less clear and dependent on the mechanical restoring force, leading to a strongly damped harmonic oscillation. In general, the homogeneity of characteristics is robust as they do not interfere with the required functionality, and deviations are reasonable and can be related to the geometry of the MEMS elements. Response times are measured below 40 µs, actuation voltages do not exceed 60 V, and the overall transmission of the ring shutter yields 53.6%, making it attractive for applications in interference microscopy.

## 5. Patent

H. Hillmer: Spiegel-Shutter Array; Patent DE 10 22,020 123 024 A1 (2020)

## Figures and Tables

**Table 1:** Transmission results in the initial fully open state. The first row resembles the measured transmission for each configuration depicted in Figure 4, whereas in the second row, the relative deviation in transmission of each ring/zone with respect to the overall device measure is calculated.

Measure	Overall	Ring A	Ring B	Ring C	Ring D	Ring E	Zone 1	Zone 2	Zone 3
Transmission in %	53.6	44.5	49.4	51.9	53.9	58.1	54.3	53.4	52.9
Relative deviation in %	0 (Ref.)	−17.0	−7.9	−3.1	0.6	8.3	1.3	−0.4	−1.3

**Table 2:** Results based on evaluating the T-V curves for rings, zones, and the overall device.

Measure|Evaluation	Overall	Ring A	Ring B	Ring C	Ring D	Ring E	Zone 1	Zone 2	Zone 3
Closing threshold in V	+46.5 −51.7	+46.5 −51.3	+46.8 −51.8	+49.1 −52.8	+49.3 −51.0	+49.9 −53.2	+47.2 −53.5	+46.2 −51.5	+46.1 −51.6
Fully closed in V	+56.0 −61.3	+55.8 −61.6	+54.6 −62.4	+59.1 −63.5	+59.1 −62.2	+60.2 −62.9	+57.7 −63.0	+57.1 −60.8	+54.4 −59.6
Opening threshold in V	+32.9 −39.0	+32.0 −39.4	+33.1 −38.4	+33.7 −38.5	+34.8 −38.2	+34.8 −38.2	+33.8 −40.4	+35.5 −40.4	+32.9 −40.2
Fully open in V	+25.9 −32.4	+23.6 −30.1	+26.2 −31.0	+26.9 −30.1	+27.6 −29.7	+27.4 −30.8	+25.3 −32.5	+24.4 −30.7	+25.9 −31.9
Closing range in V	9.5 9.6	9.3 10.3	7.8 10.6	10.0 10.7	10.7 11.2	10.3 9.7	10.5 9.5	10.9 9.3	8.3 8.0
Deviation in closing threshold in V|%	0|0 0|0	0.0|0.0 −0.4|−0.8	0.3|0.7 0.1|0.2	2.6|5.6 1.1|2.1	2.8|6.0 −0.7|−1.4	3.4|7.3 1.5|2.9	0.7|1.5 1.8|3.5	−0.3|−0.7 −0.2|−0.4	−0.4|−0.9 −0.1|−0.2
Holding gap in V	23.1 22.3	23.8 22.2	21.5 24.0	25.4 25.0	25.3 25.2	25.4 24.7	23.9 22.6	21.6 20.4	21.5 19.4

**Table 3:** Response dynamics, i.e., closing and reopening times of rings, zones and the overall device.

Response Time	Overall	Ring A	Ring B	Ring C	Ring D	Ring E	Zone 1	Zone 2	Zone 3
Closing in µs	31.7	26.4	28.5	30.3	33.0	35.2	31.9	31.6	33.9
Opening in µs	39.3	34.3	31.5	31.6	32.0	37.6	36.8	38.3	36.9

## Data Availability

The original contributions presented in this study are included in the article. Further inquiries can be directed to the corresponding author.
